# Discrimination of plant-based milk alternatives: a pilot study comparing mid and near-infrared spectroscopy

**DOI:** 10.1007/s00216-026-06737-2

**Published:** 2026-08-12

**Authors:** Stephan Freitag, Lukas Schlierenzauer, Marija Magdalena Babić, Bojan Šarkanj, David Lilek, Rudolf Krska

**Affiliations:** 1 Department of Agricultural Sciences, Institute of Bioanalytics and Agro-Metabolomics, BOKU University, Konrad Lorenz Str. 20, 3430 Tulln, Austria; 2 Department of Food Technology, University North, Trg Dr. Žarka Dolinara 1, 48000 Koprivnica, Croatia; 3 Biotech Campus Tulln, University of Applied Sciences Wiener Neustadt, Konrad Lorenz Str. 10, 3430 Tulln, Austria; 4 Department of Chemistry and Physics of Materials, Paris Lodron University Salzburg, Jakob-Haringer-Str. 2a, 5020 Salzburg, Austria; 5 Institute for Global Food Security, School of Biological Sciences, Queen’s University Belfast, University Road, Belfast, Northern Ireland BT7 1NN UK

**Keywords:** Near-infrared, Plant-based milk alternative, Partial least squares discriminant analysis, Mid-infrared, One-class, Multi-class

## Abstract

Plant-based milk alternatives (PBMAs) are expanding rapidly, motivating the development of fast, minimal-prep analytical tools for their analysis. We collected and analyzed (*n* = 154) commercial PBMAs (almond, oat, rice, soy, coconut) from Austrian and Croatian supermarkets using attenuated total reflection mid‑infrared (ATR‑MIR) and diffuse reflection near‑infrared (DR‑NIR) spectroscopy to compare classification performance across spectral regions. Spectra were acquired without notable sample preparation and processed using second-derivative transformation. Exploratory principal component analysis (PCA) and partial least squares discriminant analysis (PLS‑DA) were applied in one‑class (PLS1‑DA) and multi‑class (PLS2‑DA) modes. Comparison of using either NIR or MIR was done on the Austrian samples only, as only legacy MIR data was available for the Croatian samples. A significant difference (*p* < 0.05) in the obtained predictions was found between the usage of MIR and NIR for one-class PLS1-DA identification of oat and almond models as well as for the discrimination using the PLS2-DA multi-class approach. Cross‑validated multi‑class macro‑F1-score reached 0.90 (MIR) versus 0.81 (NIR), a F1-score of 0.98 for one-class almond classification using MIR data and 0.90 for soy one-class classification using the NIR data was obtained. The MIR models trained on Austrian data showed excellent generalization on Croatian samples, for almond, oat, rice, and soy, while coconut showed poor generalization. Overall, our results suggest that ATR‑MIR offers slightly better discrimination to DR‑NIR for PBMA classification, though NIR remains attractive for straightforward portable screening. In the future, expanding sample diversity and exploring advanced machine learning modelling could further improve performance, particularly for coconut PBMAs.

**Graphical Abstract:**

**Figure:**
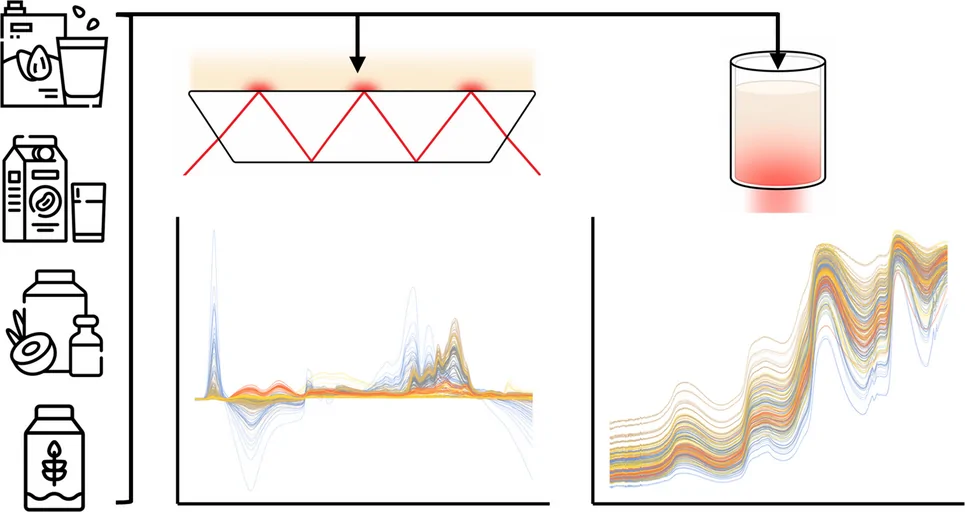

**Supplementary Information:**

The online version contains supplementary material available at https://doi.org/10.1007/s00216-026-06737-2.

## Introduction

Plant-based milk alternatives (PBMAs) are becoming more popular among Europeans and Northern American consumers [1]. This is linked to dietary as well as ethical concerns [2–4]. In the last years, a wide variety of such drinks based on different plants have entered the market [5]. These products include alternatives based on oats, almonds, soy, rice, coconut, and other plant types [2, 4]. The processing of this different plant materials is done with the goal to mimic bovine milk [3, 4] regarding the taste and appearance. The composition and nutrient density vary depending on the PBMA [4–7], having lower nutritional values than milk [4–6]. Nevertheless, due to perceived health benefits, lifestyle, and environmental and ethical concerns, it is not likely that PBMAs will disappear from the market [1, 4]. On the contrary, the diversity and market share of plant-based milk alternatives in Western countries are likely to increase in the coming years [4].

Infrared (IR) spectroscopy has been used to analyze milk and other dairy products since the 1960 s [8]. Early implementations included calibrations for protein, fat, and lactose content, while modern IR-based approaches focus on monitoring animal health, assisting cow-breeding, and detecting adulteration in dairy products [9–11]. For routine purposes, mid-IR (MIR) and near-IR (NIR) spectrometers with on-demand calibrations are available [12]. In general, both spectral regions come with advantages and disadvantages. For example, NIR allows for longer optical pathlengths, enabling more robust transmission measurements [13], while MIR provides clearer and easier to interpret chemical information [14, 15].

Compared to the use of IR spectroscopy for diary analysis, only few studies exist which explore the analysis of PBMAs. Recent studies have shown that MIR spectroscopy (MIRS) can be used to discriminate PBMAs. Christodoulou et al. have shown that attenuated total reflection (ATR)-MIRS can be used to discriminate 41 commercially available almond, soy, rice, and oat PBMAs [16]. In another study, Brito et al. collected 40 commercially available almond, soy, oat, and rice PBMAs in Portugal also using ATR-MIRS paired with chemometrics for discrimination [17]. Raman spectroscopy has also been used to discriminate between almond, cashew, macadamia, oat, pistachio, and walnut PBMAs [18].

For this study, a total of 154 different almond, oat, soy, rice, and coconut PBMAs were collected from Croatian and Austrian supermarkets. ATR-MIRS and diffuse reflection (DR)-NIR spectroscopy (NIRS) were used to analyze the samples. After spectra acquisition, principal component analysis (PCA) and partial least squares discriminant analysis (PLS-DA) were used to study the differences between these samples. To the best of our knowledge, this is the first study to analyze such a diverse sample set of PBMAs exploring both NIR and MIR spectroscopy.

## Materials and methods

### Sample collection

The PBMA samples were collected over 2 years. The Croatian samples were collected in 2024 (*n* = 38), consisting of 10 almond, 2 coconut, 11 oat, 8 rice, and 7 soy samples. The Austrian samples were collected in 2025 (*n* = 116): 24 almond, 18 coconut, 38 oat, 14 rice, 21 soy, and 1 mixed oat-almond sample. Further details on the samples are available online [19].

### Spectra acquisition

For MIR spectra acquisition, an Invenio X FT-IR spectrometer (Bruker Optics, Ettlingen Germany), equipped with a liquid nitrogen cooled mercury cadmium telluride detector and a Miracle Triple bounce ATR accessory (PIKE Technologies, Fitchburg, WI, USA) was used. One hundred microliters of the PBMA samples was pipetted on the ATR crystal. A total of 32 scans, using a spectral resolution of 4 cm^−1^, were averaged per measurement, resulting in a spectra acquisition time of ~ 2 s. Before the measurements, 100% lines were recorded as a quality control measure. The FT-IR was flushed with dry air and considered stable when a root mean square noise of 10^−5^ AU was obtained in the spectral region between 1700 and 1500 cm^−1^. For the background spectrum, water was pipetted on the ATR crystal and the spectrum recorded.

For NIR spectra acquisition, the Bruker (Bruker Optics, Ettlingen Germany) multi-purpose analyzer (MPA) FT-NIR spectrometer was used, equipped with a lead sulfide detector. Approximately 10 ml of the sample was filled in a round vial and placed in the sampling interface for DR measurements. The internal gold reflection standard was used as background. For each measurement, 64 spectral scans at a resolution of 4 cm^−1^ were averaged, resulting in a measurement time of 3 min.

Every sample was measured three times, always using a fresh aliquot. From all samples, MIR spectra were recorded whereas NIR spectra were only recorded from the Austrian samples. The MIR spectra of the Croatian and Austrian samples were recorded by a different analyst, respectively, with several weeks between the measurements on the same FT-IR. The MIR and NIR spectra can be found online [19].

### Spectra processing and chemometrics

Data analysis was done in R4.4.0 using the interpreter and development environment RStudio (Posit Software, PBC, Boston, MA, USA). The packages prospectr [20], mdatools [21], pls [22], ggpubr [23], and ggsci [24] were used for spectra processing, multivariate statistics, and plotting. The MIR spectra were cut to the fingerprint region (1800–800 cm^−1^) before further analysis. The second order gap derivative was used for NIR and MIR spectra processing as implemented in the prospectr package. For the MIR spectra, a window and segments size of 3 was used and for the NIR spectra, a window size of 5 and a segment size of 31 were used. Following spectra processing, PCA was used to explore general trends in the data set [25]. For discriminating the different types of plant-based milk alternatives, PLS-DA was used. The class labels (i.e., soy, oat, rice, coconut, almond) were converted into numerical dummy variables. Two PLS-DA approaches were explored. First five one-class (PLS1) models were tested (e.g., soy (1) vs. other (− 1)). Then, multi-class PLS2-DA using a matrix of dummy variables instead of a vector was performed [26]. Cross-validation (CV) was conducted using a stratified, grouped sevenfold strategy. For each sample, three separate aliquots (replicates) were measured. A grouped splitting strategy was implemented to ensure that all replicates of a single physical sample were assigned to the same fold, thereby preventing data leakage and optimistic bias. Furthermore, the split was stratified to reflect the overall class distribution of the data set. Given that rice PBMAs represented the smallest class in the Austrian sample set (14 different samples), a sevenfold split was chosen to guarantee that exactly two rice samples (represented by 6 replicate measurements) were assigned to the test fold in each CV loop. The same sample assignment during CV was used for the one-class and multi-class PLS-DA models developed on the MIR and NIR data. The per-fold performance of the models was then used to evaluate the use of MIR or NIR spectroscopy for PBMA discrimination. The Wilcoxon signed-rank test for paired data was performed to test the hypotheses that using MIR or NIR spectroscopy will yield different PLS-DA model performances. For the one-class PLS1-DA approach, the number of latent variables was selected using the perturbation approach [27], as implemented in the pls package. In the multi-class PLS2-DA approach, ratio plots were used to choose the number of latent variables [28]. The PLS-DA models were trained using the Austrian samples only, as NIR and MIR spectra were only available of these samples. For the PLS-DA models obtained using the MIR data, the Croatian samples were used to test the generalization of the trained models, as only for these samples historical MIR data was available.

The F1-score was used to evaluate classification performance of the one-class PLS-DA models, as this metric is robust against class imbalances as faced in this study. For the multi-class models, the macro-F1-score was used, which is simply the average of the F1-scores for the single classes (*k*). These scores were calculated as follows:

1 $$F1-\mathrm{s}\mathrm{c}\mathrm{o}\mathrm{r}\mathrm{e}= \frac{2\mathrm{T}\mathrm{P}}{2\mathrm{T}\mathrm{P}+\mathrm{F}\mathrm{P}+\mathrm{F}\mathrm{N}}$$

2 $$\mathrm{M}\mathrm{a}\mathrm{c}\mathrm{r}\mathrm{o}-F1-\mathrm{s}\mathrm{c}\mathrm{o}\mathrm{r}\mathrm{e}= \frac{1}{k}\sum_{i=1}^{k}{{F}_{1}}_{i}$$

where true positive (TP) is a sample assigned correctly to its class by the PLS-DA model. False negative (FN) is a sample not assigned to its class, and false positive (FP) a sample assigned to the wrong class. The R code used for plotting and data analysis can be found online [19].

## Results

**Fig. 1:**
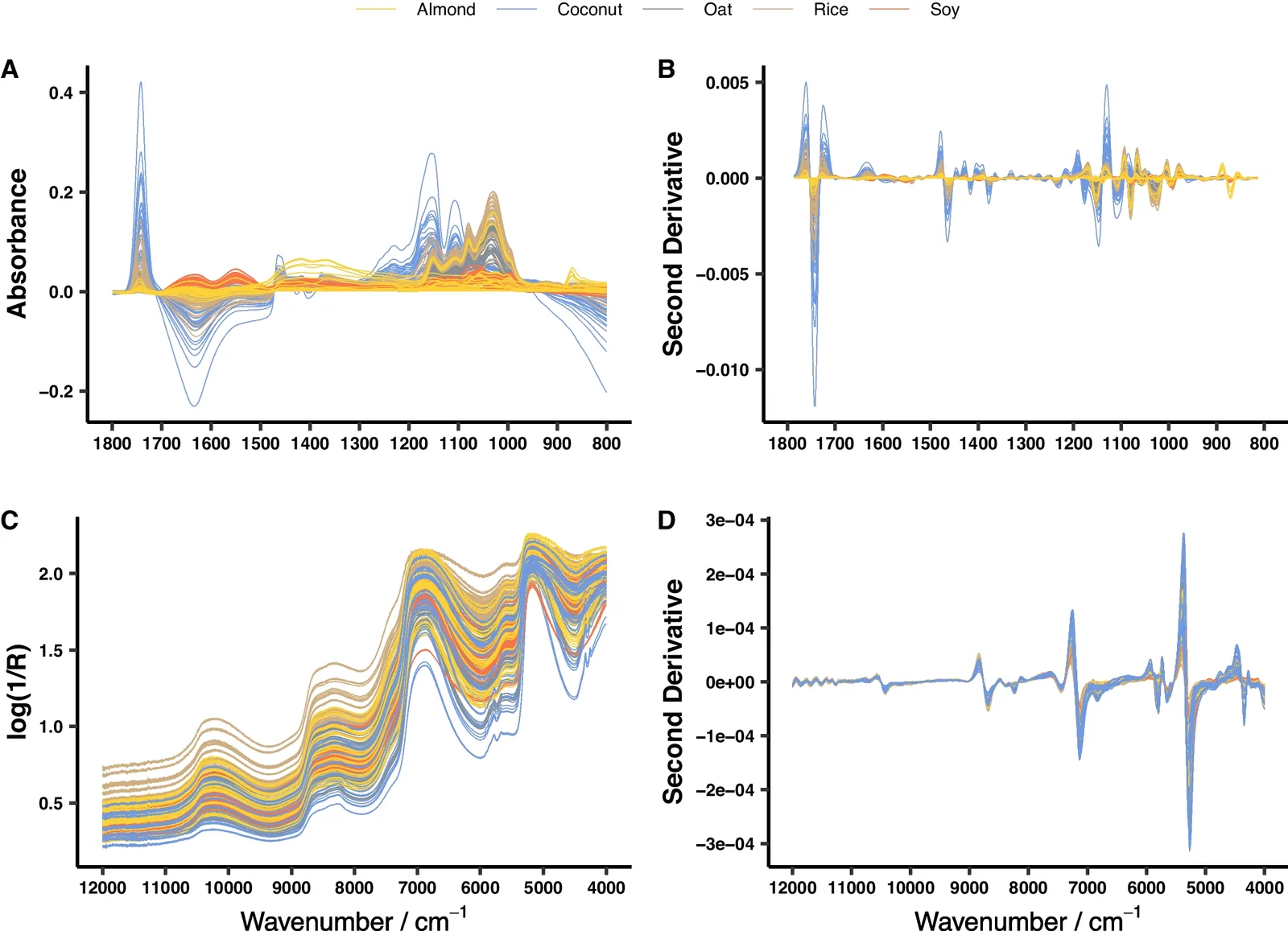
Mid-infrared (**A**) and near-infrared (**C**) spectra of the plant-based milk alternatives and the corresponding second derivative (**B**, **D**)

For processing the MIR and NIR spectra, the second derivative was chosen to resolve overlapping spectral features and also to get rid of baseline drifts. The raw and processed IR spectra can be seen in Fig. 1.

**Fig. 2:**
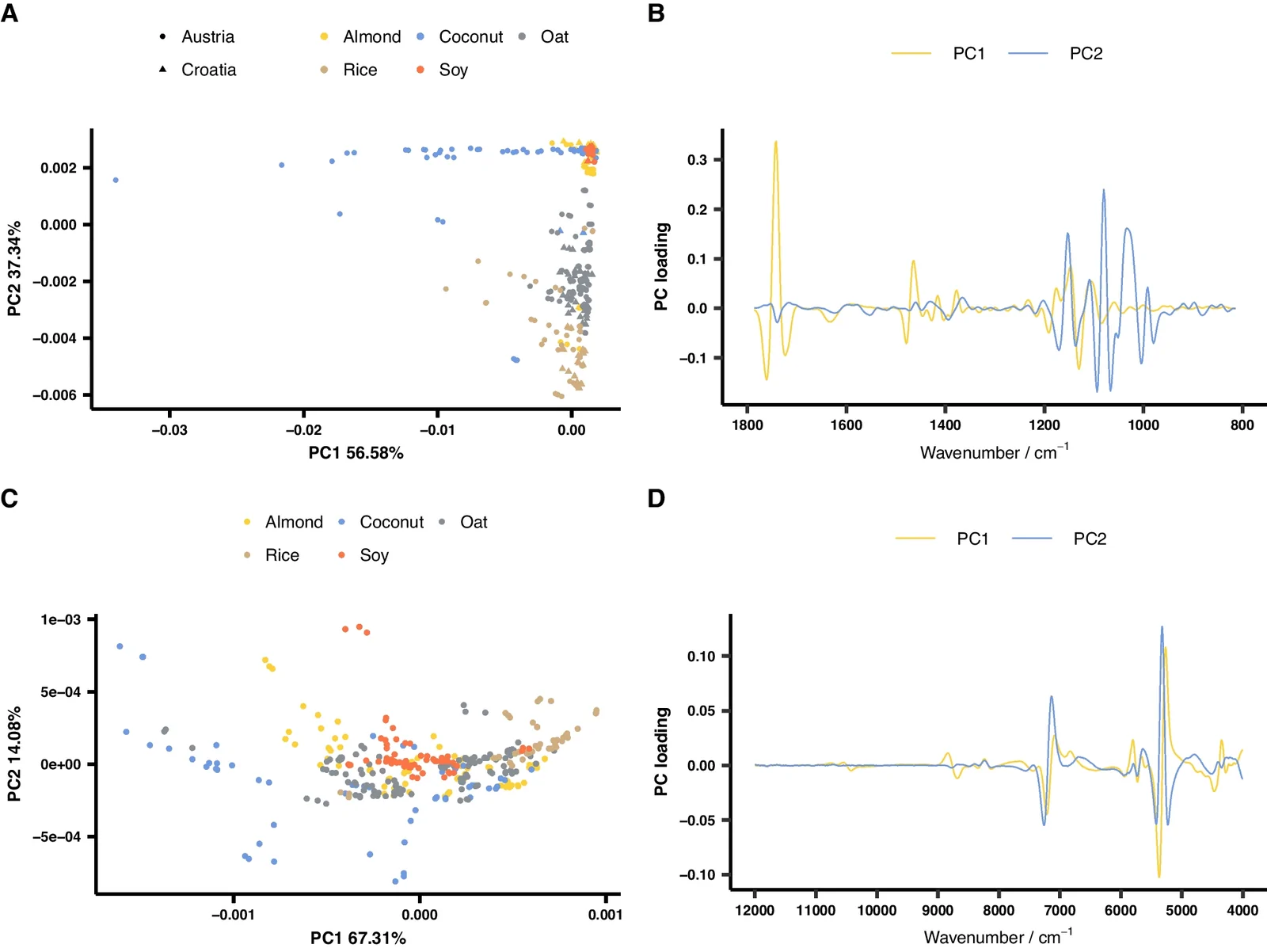
Results of principal component analysis. Score (**A**) and loading plot (**B**) obtained using the mid-infrared data. Score (**C**) and loading plot (**D**) using the near-infrared data. PC, principal component

The spectra processing strategy was guided by exploratory data analysis using PCA, as well as the results obtained during CV of the PLS-DA models on the Austrian samples. The obtained score and loading plots of the PCA can be seen in Fig. 2.

**Fig. 3:**
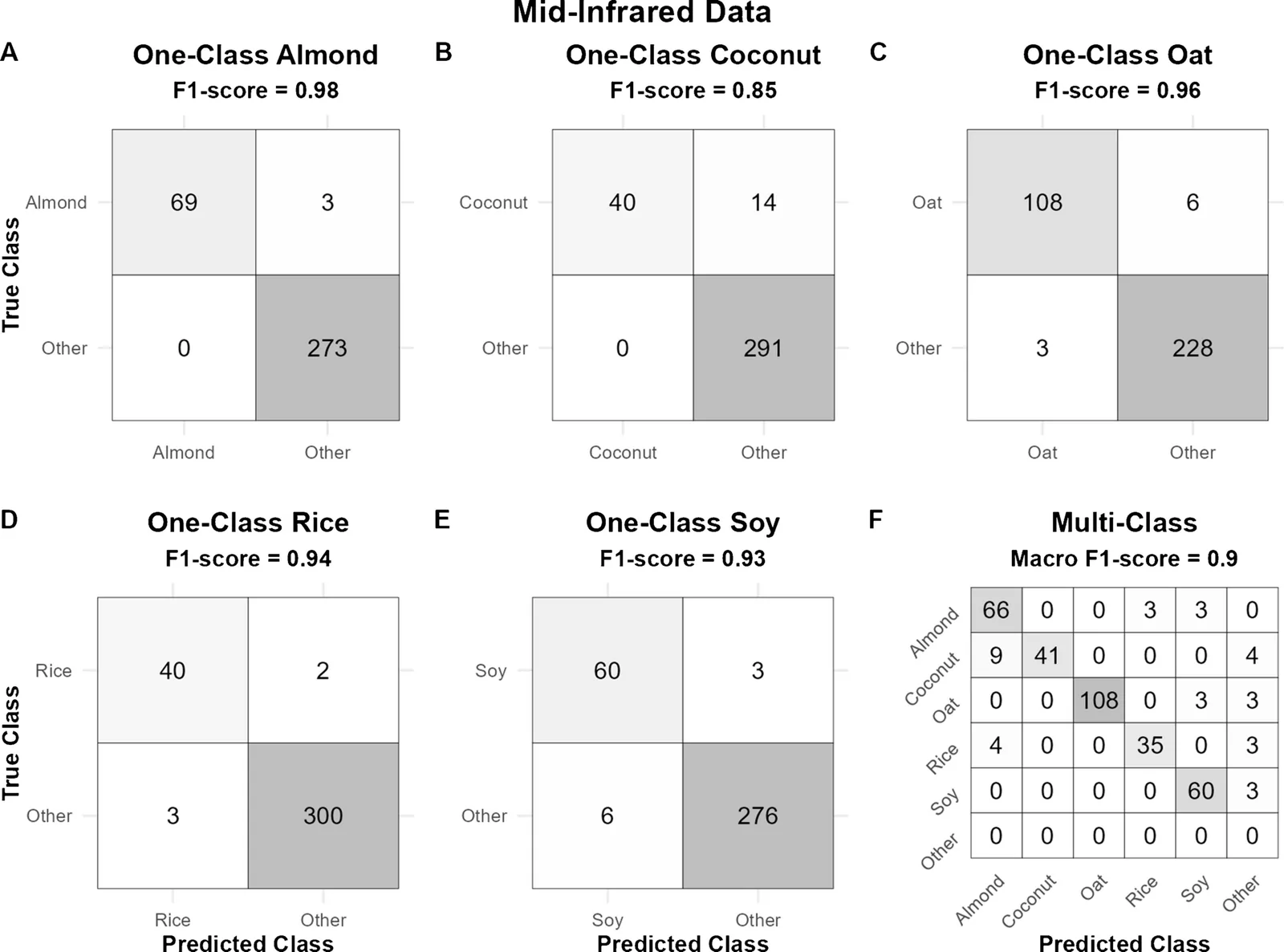
Confusion matrices (CMs) showing the classification performance using partial least square discriminant analysis obtained using cross-validation on the mid-infrared spectra of the plant-based milk alternatives (PBMAs) sourced in Austria. **A**–**E** CMs of the one-class models for the respective class of PBMA. **F** CM of the multi-class model

Following exploratory data analysis, discriminant analysis was done using PLS. The results of LV selection can be found in Supporting Figs. 1 and 2. For both data sets, one-class PLS1-DA (e.g., almond vs. other) and multi-class PLS2-DA were used to discriminate between the different PBMA types. The results obtained using CV on the MIR spectra of the PBMAs collected in Austria are shown in Fig. 3.

**Fig. 4:**
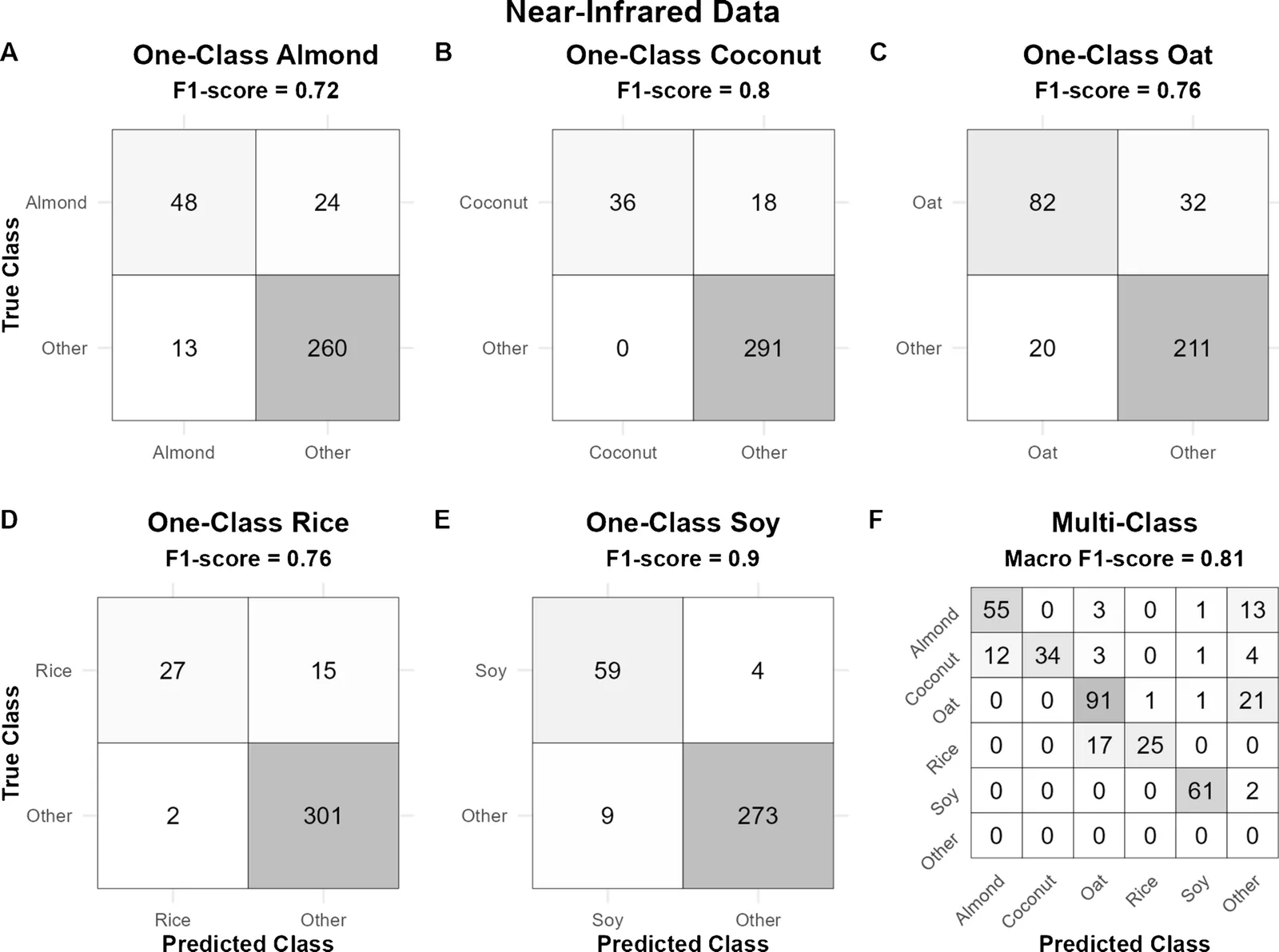
Confusion matrices (CMs) results using partial least square discriminant analysis (PLS-DA) obtained using cross-validation on the near-infrared spectra of the plant-based milk alternatives (PBMAs) obtained in Austria. **A**–**E** CMs of the one-class PLS1-DA models for the respective PBMA. **F** CM of the multi-class PLS2-DA approach

For the MIR data set after choosing the number of latent variables using CV on the Austrian samples, the generalization performance of the models was further evaluated using the Croatian samples (see Supporting Fig. 3). The classification performance obtained by PLS-DA during CV using the NIR data can be seen in Fig. 4.

**Fig. 5:**
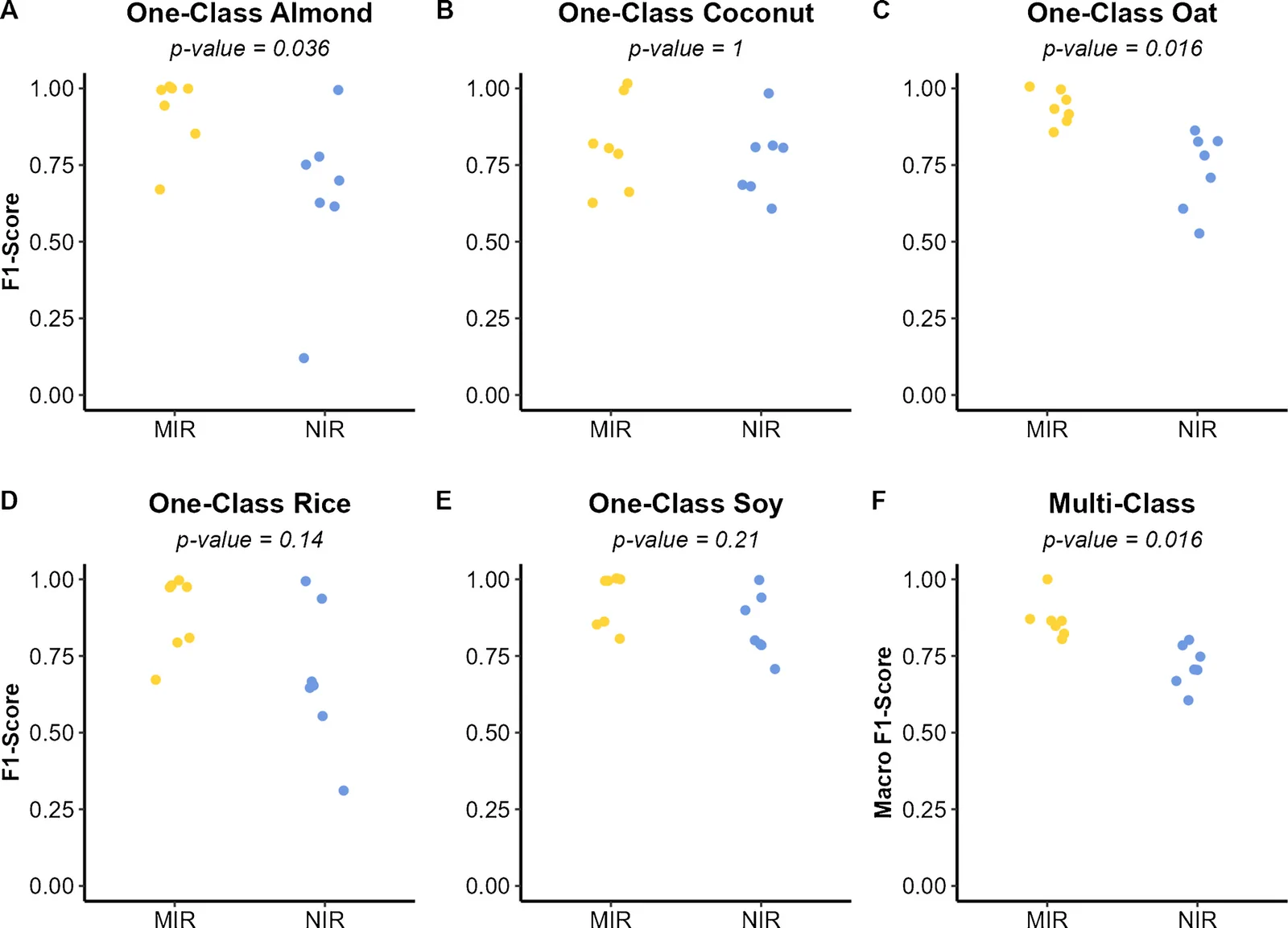
Comparison of partial least squares discriminant analysis (PLS-DA) model performance using either mid-infrared (MIR) or near-infrared (NIR) data using fold wise F1-scores obtained during 7-fold cross validation. **A**-**E** one-class PLS1-DA.** F** multi-class PLS2-DA. The *p*-values were obtained using the Wilcoxon signed-rank test

After model training, the performance of the PLS-DA models was compared using the fold-wise F1-scores obtained during CV; the results are found in Fig. 5.

## Discussion

The obtained IR spectra of the PBMAs show relatively sharp MIR absorption bands compared to broader NIR bands (see Fig. 1). The DR-NIR spectra contain information linked to the scattering properties of the PBMAs arising from the suspended particles. This leads to a slope in the baseline and non-linearities, in addition to the chemical information, mainly from the absorption of O–H, C–H, and N–H functional groups [29]. In contrast to the observed baseline slope in the DR-NIR spectra, the ATR-MIR spectra have a rather flat baseline, underscoring the robustness of the technique against scatter effects. For the MIR spectra, clear differences between the different types of PBMAs can be observed. The MIR spectra of the coconut PBMA are dominated by absorption features linked to fat, such as the triglyceride ester C = O stretch at 1750 cm^−1^, the CH_2_/CH_3_ deformations at 1460 cm^−1^ and 1380 cm^−1^, as well as the C-O ester stretches at around 1150 cm^−1^ [14, 15]. The cereal-based PBMAs (i.e., rice and oat) show strong C–O/C–O–C ring and glycosidic vibration features in the carbohydrate region (1150–950 cm^−1^), while in the soy PBMA spectra, the strongest absorbance bands linked to proteins (1700–1500 cm^−1^) in the amide I (1700–1600 cm^−1^) and amide II region (1600–1500 cm^−1^) among the different types of PBMAs are found [14, 15]. Almond PBMA spectra exhibit the weakest absorbance features of all PBMAs, with notable differences in the carbohydrate region. The apparent negative absorbance (1700–1500 cm^−1^), mainly seen in the spectra of coconut PBMAs, is caused by using water as the background spectrum. Rice and coconut PBMAs seem to contain the least amount of water, leading to the strong negative bands around 1640 cm^−1^ and below 950 cm^−1^ [15].

Differences in the NIR spectra of the different types of PBMAs are less pronounced compared to the MIR spectra. The NIR spectra are dominated by water because a reflection standard was used as the background. This results in the broad O–H first overtone near 6900 cm^−1^ and a very strong combination band around 5200 cm^−1^, with the weaker O–H second overtone near 10,300 cm^−1^ [13]. Coconut PBMA shows the most intense lipid signatures, with pronounced C–H second overtones between 8000 and 8200 cm^−1^ and strong first overtones around 5800 to 5600 cm^−1^, as well as combination features between 4000 and 4300 cm^−1^ [13]. Soy displays the clearest protein fingerprints, notably the N–H first overtone in 6700–6400 cm^−1^ and amide/N–H combinations near 5000 to 4800 cm^−1^ [13]. In the spectra of oat and rice PBMAs, absorption features linked to carbohydrate-related O–H structure on the higher wavenumber side of the water band (7300 to 7000 cm^−1^) can be found, with weaker lipid and protein bands than coconut and soy, respectively [13]. Almond absorption features are, as in the MIR spectra, less pronounced compared to the other PBMAs. For both MIR and NIR spectra, in general, coconut is distinguished by lipids, soy by proteins, and oat/rice by carbohydrates.

For the PCA on the MIR spectra (see Fig. 2), principal component (PC)1 largely contains information linked to the lipid ester and CH deformation vibrations. The strongest loading in PC1 is the triglyceride C = O stretch near 1750 cm^−1^, followed by CH₂/CH₃ deformations at 1460 cm^−1^ and 1380 cm^−1^ and C–O ester stretch at 1150 cm^−1^ [15]. In general, coconut is predominantly pushed to one side of the score plot while still spanning the entire PC1. PC2 emphasizes protein and carbohydrate features. The loading around the amide II spectral region explains the aggregation of protein-rich PBMAs to one side of PC2, while multiple features in the carbohydrate fingerprint region (1150–950 cm^−1^) position oat/rice across PC2 but separately from almond and soy. Overall, PCA using the MIR spectra separates PBMAs by composition rather than country of origin, as the samples from Austria and Croatia show strong within-class overlap. Therefore, it was decided to use the Croatian sample to test the generalization of the MIR-based models.

Differences in the NIR spectra of the different types of PBMAs are less pronounced, and consequently the clusters found in the PCA score plots are less distinct and more overlapping (see Fig. 2). PC1 of the PCA using the NIR data reflects the lipid-water contrast. Strong negative as well as positive loadings at C–H first overtones (~ 5800 and ~ 5700 cm^−1^) and C–H combinations (4700–4300 cm^−1^) oppose the dominant water bands at ~ 6900 and ~ 5200 cm^−1^ [13]. Consequently, coconut scores negative on PC1 (high lipid), whereas rice tends toward a positive PC1 (higher relative water/carbohydrate content). PC2 captures protein and carbohydrate differences, with loadings around the N–H first overtone (6700–6400 cm^−1^) and amide combinations (~ 5000 to 4800 cm^−1^) giving soy slightly higher PC2 scores.

When training PLS-DA models on either the NIR or MIR data, good classification performance was found during CV (see Figs. 3 and 4). During training of the PLS-DA models using the MIR spectra, most samples were correctly predicted, with almond, oat, soy, and rice displaying excellent separation from other PBMAs. Only a few samples were assigned to the wrong class (see Fig. 3 and Supporting Fig. 4). Discriminating coconut PBMA appears to be more challenging when using MIR data than NIR data. The multi-class MIR-based model confirms high overall accuracy but reveals that some samples could not be assigned to any class at all. Following CV, the generalization of the PLS-DA models using the ATR-MIR spectra was tested using the Croatian samples (see Supporting Fig. 3 and Supporting Fig. 5). For the one-class almond and rice model, a slight decrease was observed, which is usually expected during external validation [30, 31]. The increase in the performance for the discrimination between soy and oat in the Croatian sample set (F1-score = 1) and the complete failure for coconut (F1-score = 0) using the one-class models indicates that PBMAs collected in Croatia might not fully capture the diversity found in PBMAs collected in Austria. This is underlined by the score plot seen in Fig. 2A, where the two Croatian coconut samples show no overlap with the Austrian samples, while the oat and soy samples show strong overlap. At the time when access to the NIR device was available, the Croatian samples were not available anymore; generalization testing could therefore only be carried out for the MIR-based PLS-DA models. This limits the full evaluation of the NIR-based PLS-DA models, compared to the MIR models and thus CV performance on the Austrian samples was used for comparison.

When using the DR-NIR data to discriminate between the PBMAs collected in Austria, good discriminative performance was also found during CV. For the one-class PLS1-DA as well as the multi-class PLS2-DA model, a notable number of samples was assigned to the wrong class (see Fig. 4 and Supporting Fig. 6), compared to the MIR-based models (see Fig. 3 and Supporting Fig. 4). This is underlined by the apparent lower F1-scores obtained for the models based on NIR data (see Fig. 4). Comparing the fold-wise predictions obtained during CV using NIR and MIR data with a paired Wilcoxon signed-rank test showed that choosing either MIR or NIR spectroscopy led to a significant difference (*p* < 0.05) for discriminating almond (*p* = 0.036) and oat (*p* = 0.016) as well as for the multi-class (*p* = 0.016) PLS-DA models (see Fig. 5). For discriminating coconut, soy, and rice PBMAs using one-class models, no significant difference was found; thus, neither NIR nor MIR spectroscopy is advantageous for those classes. Therefore, results presented in this study suggest the use of MIR rather than NIR for the discrimination of PBMAs using a multi-class PLS2-DA approach. Yet for future studies, the NIR region should not be neglected, as NIR technology is unmatched in its portability [32]. It is evident that portable IR holds great promise in food safety and adulteration screening [33, 34]. The ever-shrinking size and price of portable NIR spectrometers [35] might even lead to devices available to consumers of PBMAs.

It has previously been reported that imbalanced sample sets are challenging for PLS-DA [36, 37]. However, using a one-class PLS1-DA approach, leading to imbalanced data sets, we observed excellent performance in cross-validation (CV) and for the MIR-based models on a held-out test set without addressing the class imbalance (Figs. 3 and 4 and Supporting Figs. 3, 5). The good performance found in this study may be linked to clearer class separation than in prior research.

In general, no significant difference in the performance between PLS1-DA and PLS2-DA was identified neither when using the NIR nor MIR data for discriminating PBMAs (see Supporting Fig. 7).

The underexplored character of infrared spectroscopy in food science [38] is underlined by the fact that only a few studies have investigated PBMAs with MIR. The good classification performance of this study using the MIR spectra is in good agreement with previous studies using the same spectral region [16, 17]. In contrast to this previous research, we report a simpler and faster sample-handling workflow, without any sample treatment such as the lyophilization reported before. Furthermore, we show that excellent classification performance can be achieved for soy, almond, oat, and rice PBMAs using a diverse range of samples collected in two different countries, as underlined by the reported F1-scores (see Fig. 3 and Supporting Fig. 3, 5). Surprisingly, there are no studies using NIRS for PBMAs discrimination. Nevertheless, NIR has been used for the authentication of plant-based protein powders and to detect possible adulteration [39, 40]. In general, IR-based authentication and adulteration detection are becoming more popular for plant-based protein sources [41]. Therefore, the results reported here can be considered encouraging for using NIR spectroscopy for PBMA analysis.

For this study, a rather simple chemometric data analysis strategy has been used to facilitate interpretability and comparability of the two spectral regions. Yet better discrimination performance might be obtained by exploring more complex data analysis strategies including non-linear machine learning and different spectra preprocessing [42, 43]. Therefore, other scientists are encouraged to extend the data set published alongside this study [19], and also to explore it in their research with other machine learning and chemometric approaches.

## Conclusion

A wide variety (*n* = 154) of PBMAs based on soy, almond, oat, rice, and coconut have been collected from Austrian and Croatian supermarkets. Samples from both countries were analyzed using ATR-MIR, and the Austrian samples were also analyzed using DR-NIR. For both spectral regions, spectra were acquired without notable sample preparation. Our results obtained during CV indicate that PLS-DA models trained on ATR-MIR data perform slightly better than those trained on DR-NIR data. We established both one-class PLS1-DA and multi-class PLS2-DA models and found no notable differences between the two approaches. Among the different PBMA classes, coconut-based products was the only class which might be better discriminated using DR-NIR than ATR-MIR. Further research should focus on expanding the sample pool, testing different spectra processing and data analysis strategies as well as on exploring the use of portable NIR devices.

## Supplementary Information

Below is the link to the electronic supplementary material.

Supplementary file1 (PDF 688 KB)

## Data Availability

The infrared data, sample description, and R code used for this study are available online [19].

## Declarations

### Artificial intelligence usage

The authors used the large language models GPT5.5 and Gemini 3.5 flash 5 for improving the clarity of the text and for R code curation. The model GPT Image 2 was used to generate the MIR and NIR icons in the table of content graph. Access to these models was provided by the ACOmarket-Project Academic AI.
