# Supplementary material for: Discrimination of plant-based milk alternatives: a pilot study comparing mid and near-infrared spectroscopy
Source: Anal Bioanal Chem. 2026 Aug 12;418(23):7903–12. doi: 10.1007/s00216-026-06737-2 (PMC13744567; doi:10.1007/s00216-026-06737-2)
Supplement: Supplementary file 1 [file 216_2026_6737_MOESM1_ESM.pdf]

Supporting Information

Discrimination of plant-based milk alternatives: A pilot study comparing mid and near-infrared spectroscopy

Stephan Freitag,[1*] Lukas Schlierenzauer,[1] Marija Magdalena Babić,[2] Bojan Šarkanj,[2] David Lilek,[3,4] Rudolf Krska[1,5]

1 BOKU University, Institute of Bioanalytics and Agro-Metabolomics, Department of Agricultural Sciences, Konrad Lorenz Str. 20, 3430 Tulln, Austria

2 Department of Food Technology, University North, Trg dr. Žarka Dolinara 1, 48000 Koprivnica, Croatia

3 Biotech Campus Tulln, University of Applied Sciences Wiener Neustadt, Konrad Lorenz Str. 10, 3430 Tulln, Austria

4 Department of Chemistry and Physics of Materials, Paris Lodron University Salzburg, Jakob-Haringer-Str. 2a, 5020 Salzburg, Austria

5 Institute for Global Food Security, School of Biological Sciences, Queen's University Belfast, University Road, Belfast, BT7 1NN, Northern Ireland, United Kingdom

* corresponding author: Stephan.Freitag@boku.ac.at

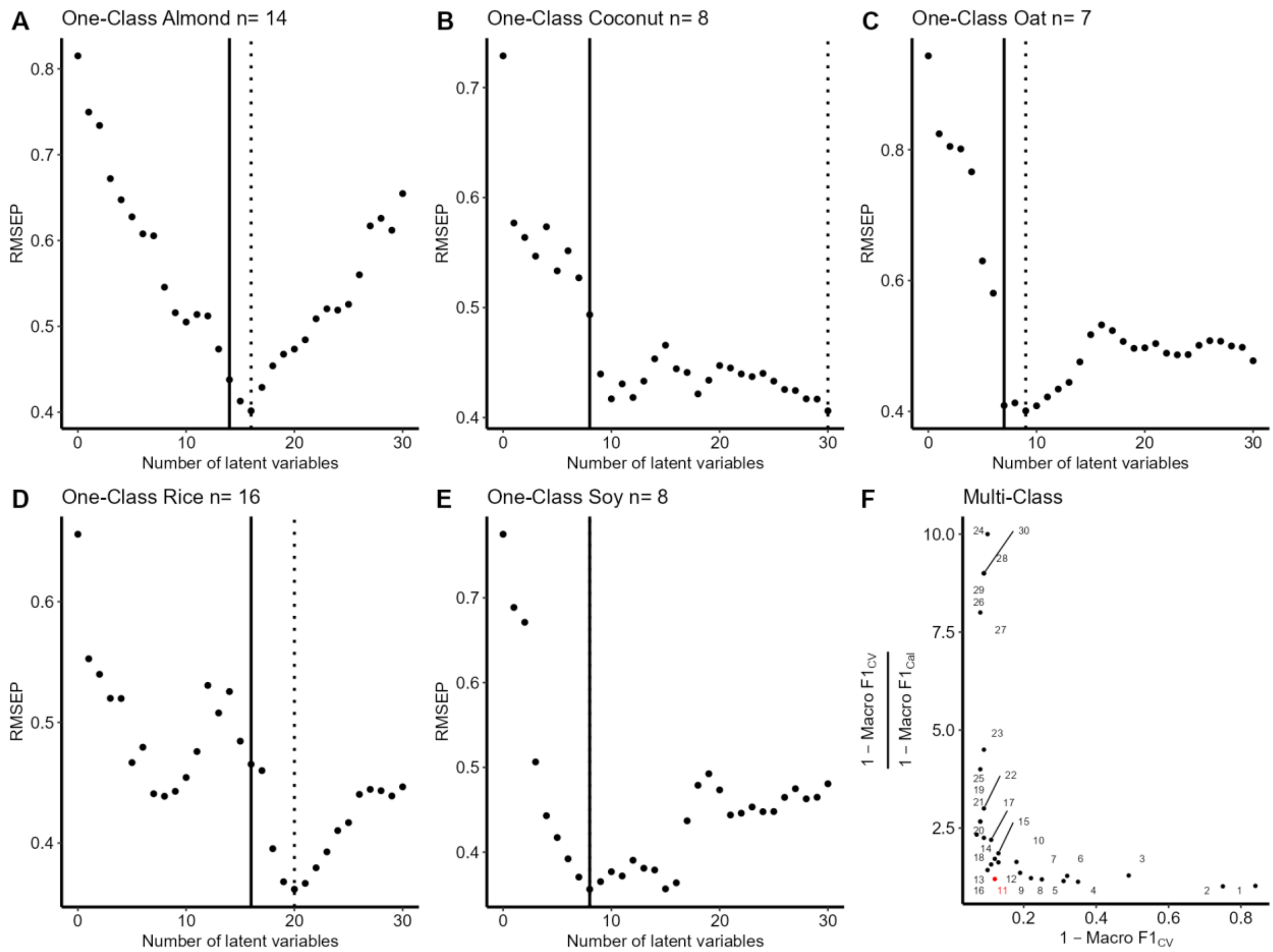

Supporting Figure 1. Results obtained for latent variable (LV) selection during cross-validation (CV) of the partial least square discriminant models using the mid-infrared data. A-E: Root mean squared error of prediction (RMSEP) versus the number of LVs for the one class models. Dotted line: minimum of the RMSEP, black line: number of LVs chosen using the permutation approach. F: Ratio plot for selecting the number of LVs of the multi class model.

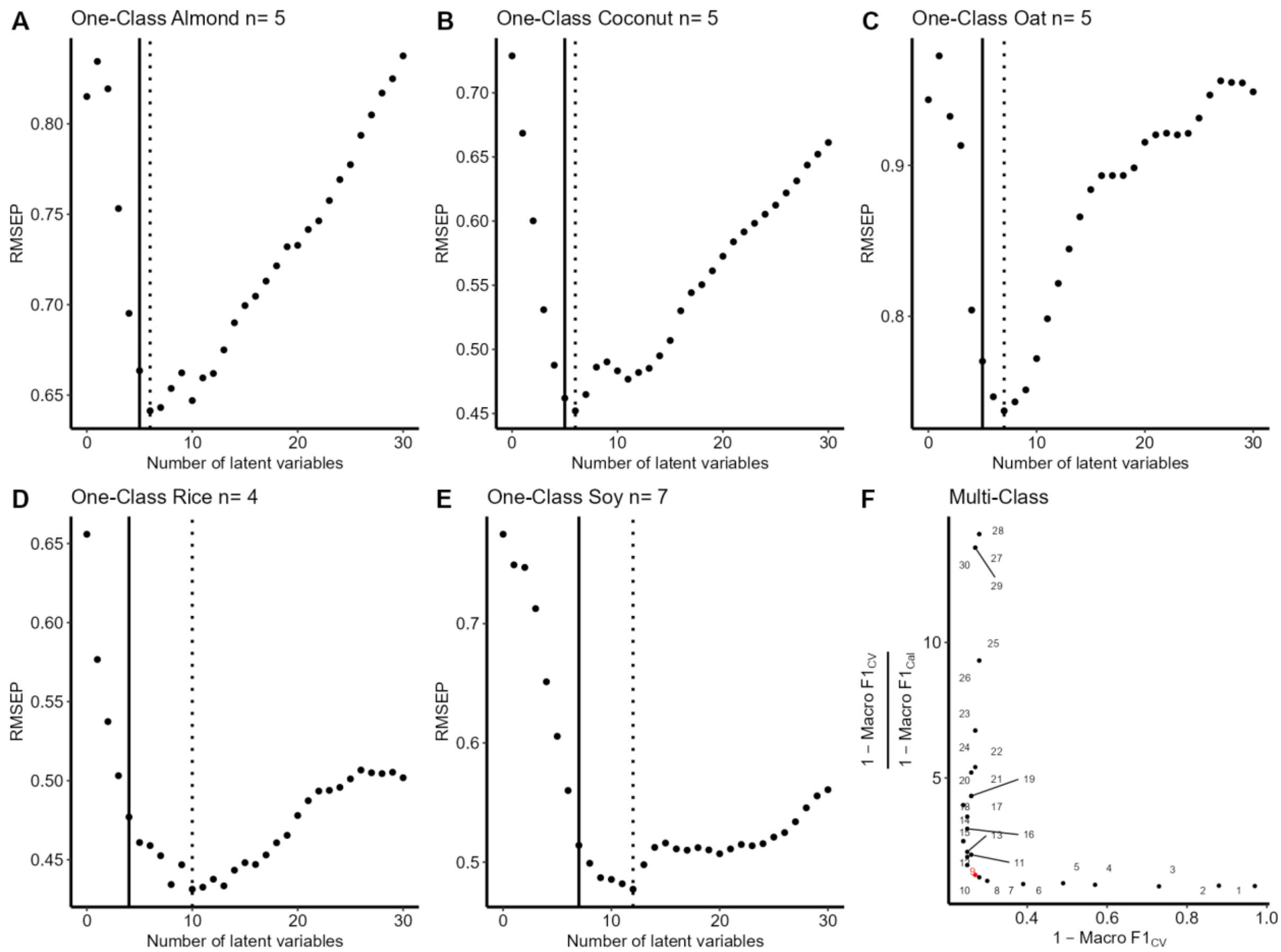

Supporting Figure 2. Results obtained for latent variable (LV) selection during cross-validation (CV) of the partial least square discriminant models using the near-infrared data. A-E: Root mean squared error of prediction (RMSEP) versus the number of LVs for the one class models. Dotted line: minimum of the RMSEP, black line: number of LVs chosen using the permutation approach. F: Ratio plot for selecting the number of LVs of the multi class model.

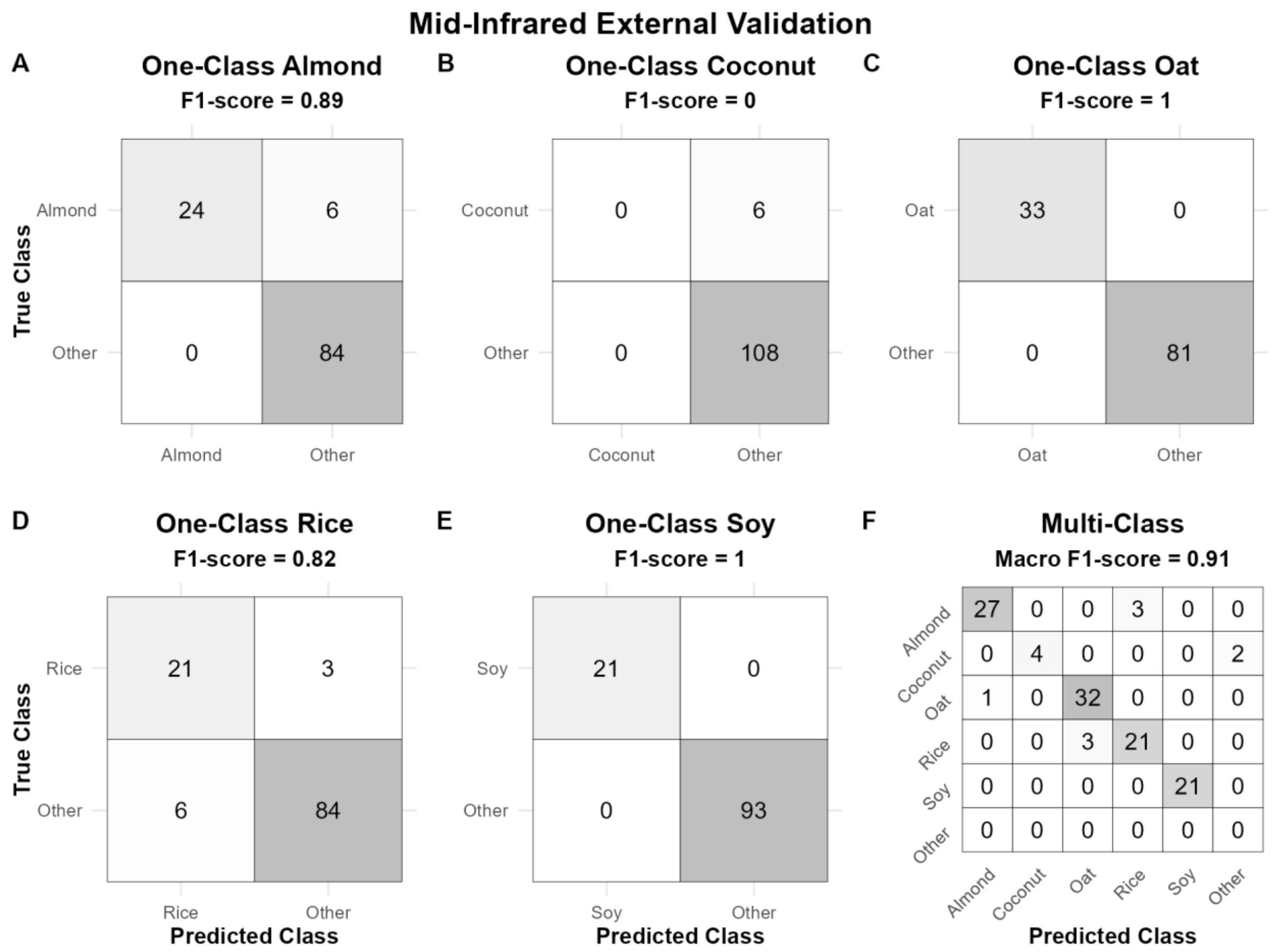

Supporting Figure 3: Confusion matrices (CM) showing the classification performance using the partial least square discriminant analysis models trained on the mid-infrared spectra of the plant-based milk alternatives (PBMAs) sourced in Austria and tested on the spectra of the Croatian samples. A-E: CM of the one class models for the respective class of PBMA, F: CM of the multi-class model.

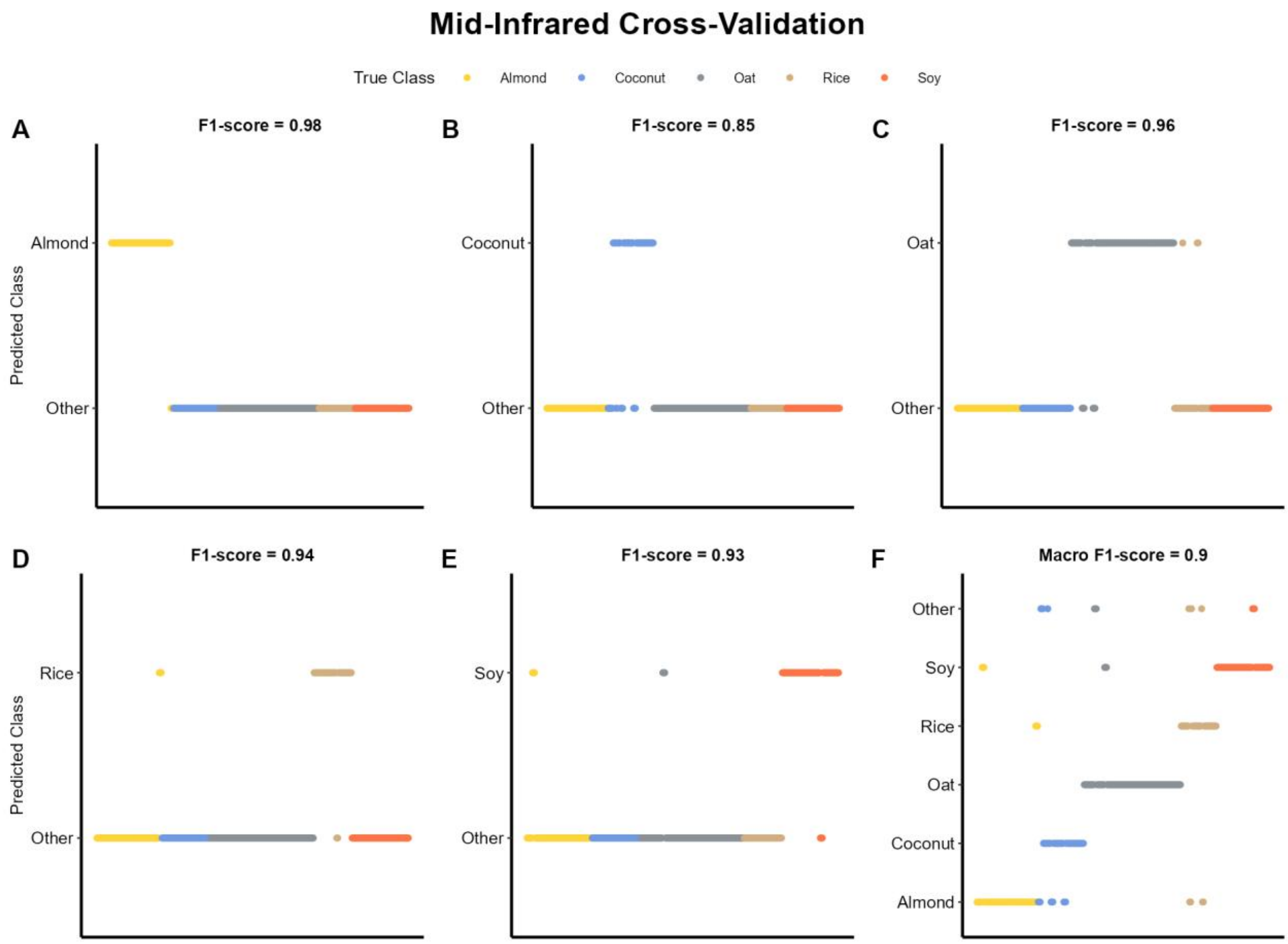

Supporting Figure 4: Classification results using partial least square discriminant analysis obtained using cross-validation on the mid-infrared spectra of the plant-based milk alternatives (PBMAs) obtained in Austria. A-E results of the one class models for the respective PBMA, F: results of the multi-class model.

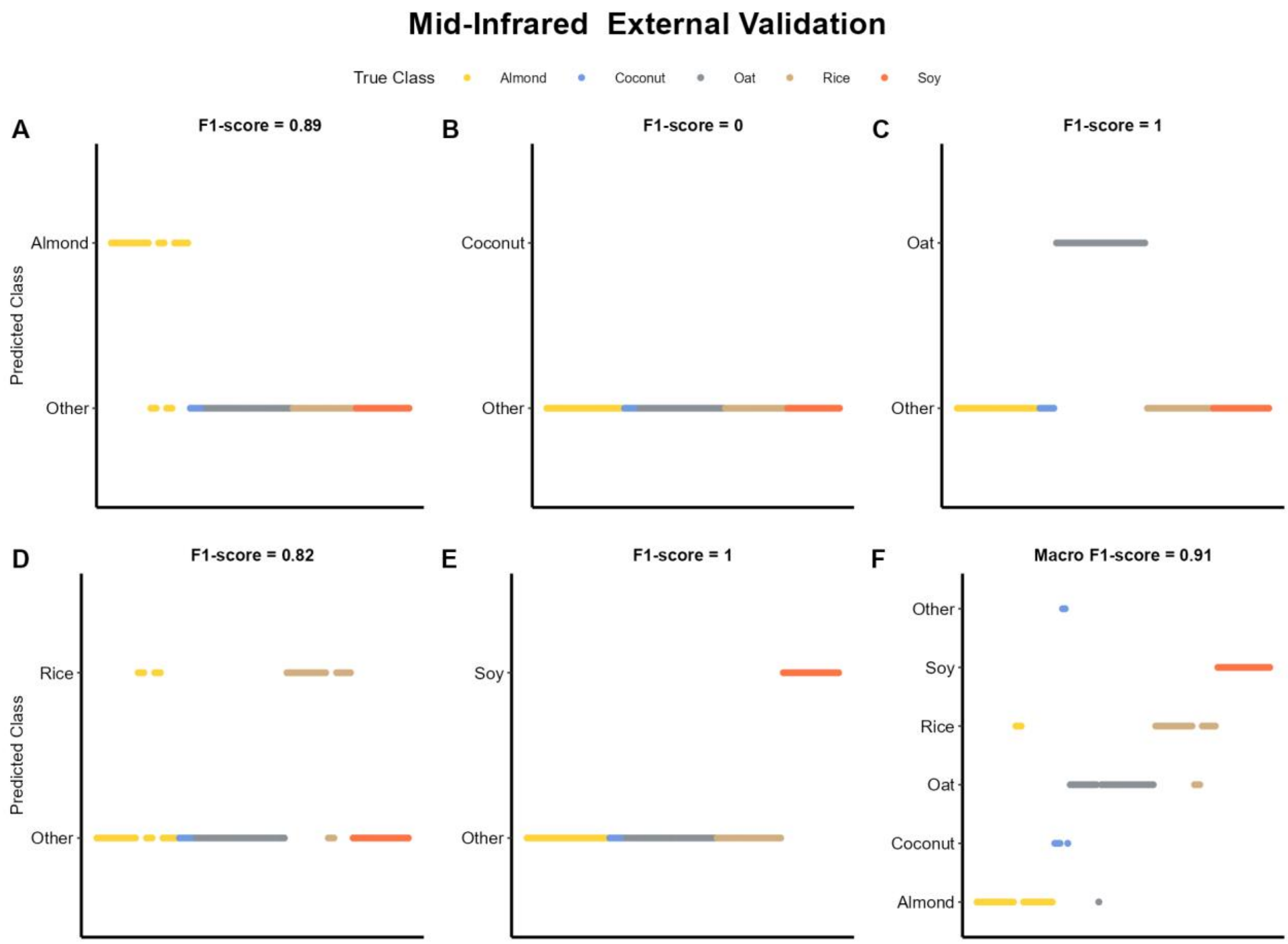

Supporting Figure 5: Classification results using partial least square discriminant analysis (PLS-DA) obtained using the mid-infrared spectra of the plant-based milk alternatives collected in Croatia using the models trained on the Austrian samples. A-E: Results of the one-class PLS1-DA models for the respective PBMA, F: Results of the multi-class PLS2-DA model.

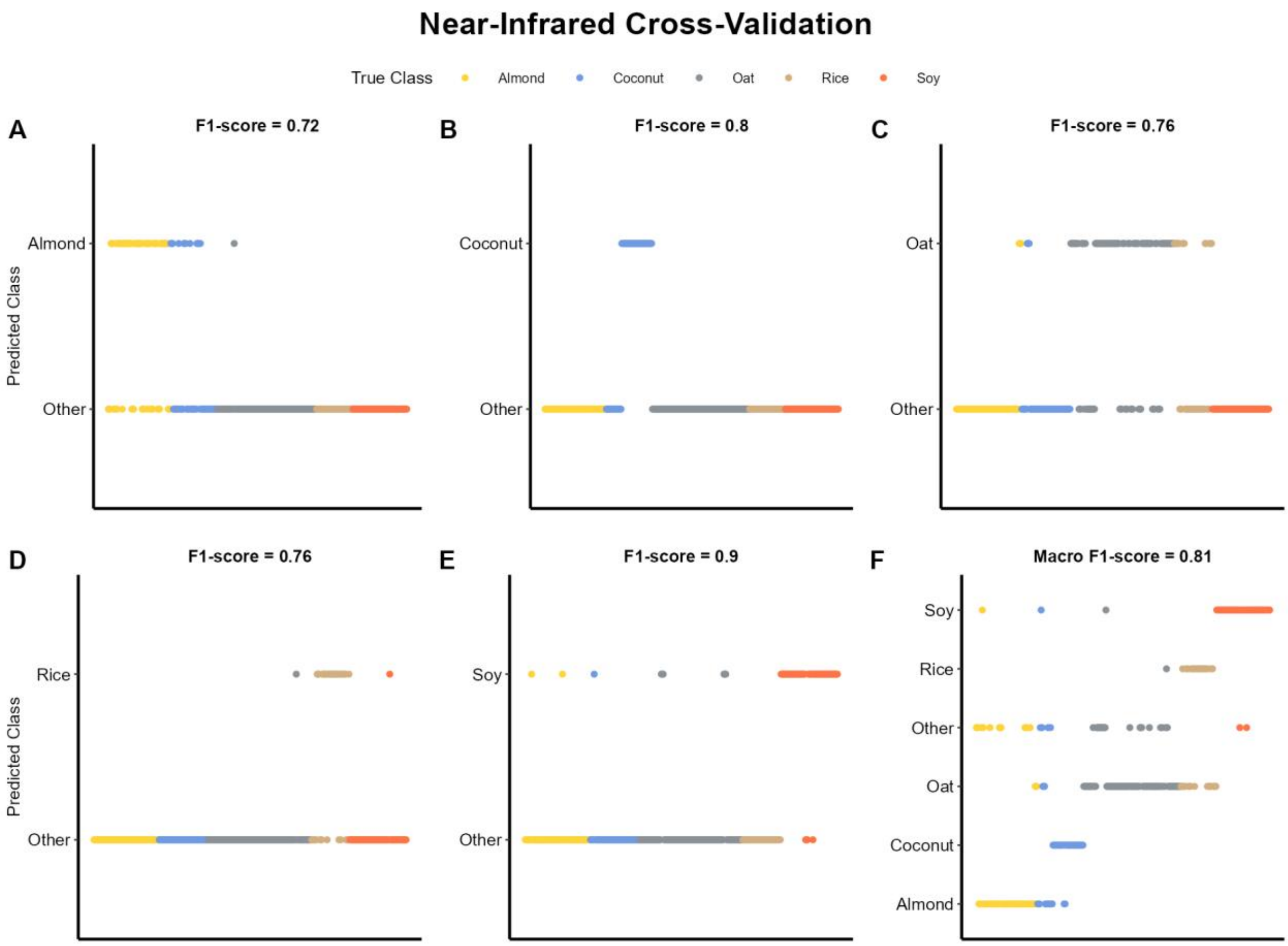

Supporting Figure 6: Classification results using partial least square discriminant analysis (PLS-DA) obtained using cross-validation on the near-infrared spectra of the plant-based milk alternatives (PBMAs) obtained in Austria. A-E results of the one class PLS1-DA models for the respective PBMA, F: results of the multi-class PLS2-DA approach.

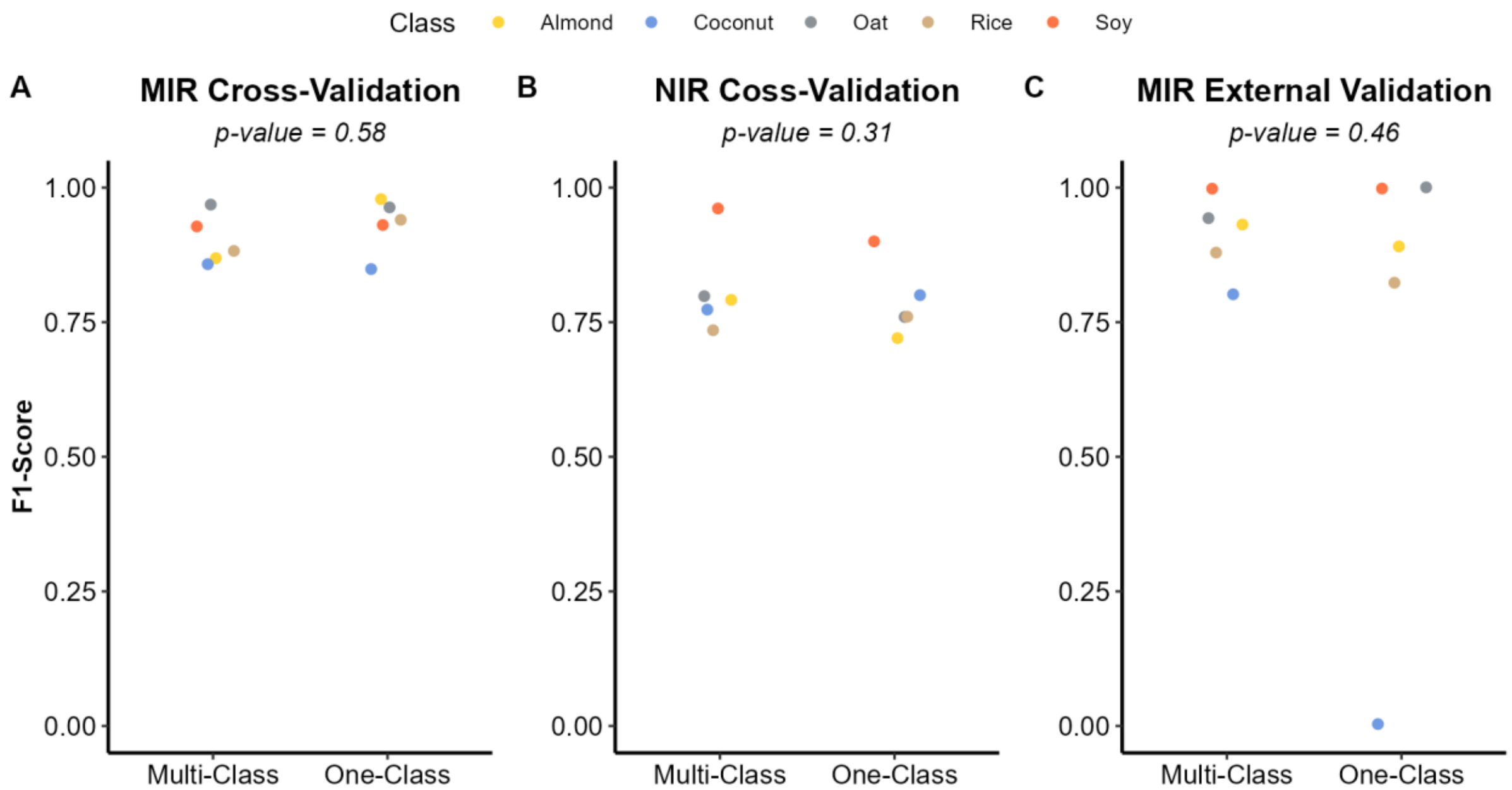

Supporting Figure 7: Comparison between one-class and multi-class partial least square discriminant analysis model performance using different data sets. MIR = mid-infrared; NIR = near-infrared
